# Identification of Biomarkers Associated with *Phyllosticta citricarpa* Tolerance

**DOI:** 10.3390/molecules29153582

**Published:** 2024-07-29

**Authors:** Puseletso O. J. Tswaai, Wilma A. Augustyn, Thierry Regnier, Wilma du Plooy

**Affiliations:** 1 Department of Chemistry, Tshwane University of Technology, P.O. Box 680, Pretoria 0001, South Africa; tswaaipoj@gmail.com; 2 Department of Biotechnology and Food Technology, Tshwane University of Technology, P.O. Box 680, Pretoria 0001, South Africa; regniert@tut.ac.za; 3 Citrus Research International, P.O. Box 28, Mbombela 1200, South Africa; wilma@cri.co.za

**Keywords:** antifungal activity, biomarkers, metabolomic profiling, *Phyllosticta citricarpa*, susceptibility

## Abstract

Citrus black spot (CBS) is a fungal disease caused by *Phyllosticta citricarpa* Kiely, (McAlpine Van der Aa), with most cultivars being susceptible to infection. Currently, disease control is based on the application of protective fungicides, which is restricted due to resistance, health and environmental concerns. Although using natural products for disease management is gaining momentum, more advances are required. This study obtained the metabolic profiles of the essential oil and cuticular waxes of two citrus cultivars with a varying susceptibility to CBS infection using gas chromatography–mass spectrometry. A multivariate data analysis identified possible biomarker compounds that contributed to the difference in susceptibility between the two cultivars. Several identified biomarkers were tested in vitro for their antifungal properties against *P. citricarpa*. Two biomarkers, propanoic acid and linalool, were able to completely inhibit pathogen growth at 750 mg/L and 2000 mg/L, respectively.

## 1. Introduction

Citrus fruits are among the world’s largest fruit crops, grown in several tropical and subtropical countries [1], and are one of the most widely traded horticultural commodities [2]. As the second largest earner of foreign income in terms of agricultural exports in South Africa, the citrus industry contributes significantly to the South African economy [3]. In general, citrus trees prefer sandy or loamy soils with good drainage and tolerate slightly acidic to slightly alkaline conditions. However, citrus trees have proven to be very adaptable to different environments and can sustain a high productivity as long as sufficient irrigation and nutrients are provided [4]. Therefore, citrus are grown in most regions in South Africa, in climates ranging from hot and dry to Mediterranean, wet and cool conditions [5]. However, citrus producers in South Africa face various pre- and postharvest challenges, such as management inefficiencies, susceptibility to pests and diseases and environmental difficulties [6,7]. Citrus black spot (CBS), which is caused by the fungus *Phyllosticta citricarpa* (McAlpine) Aa, has a significant negative impact on the industry, including increased production costs and the loss of export revenue [8]. *P. citricarpa* infections have been recorded in several countries but have not been observed in Europe and the Mediterranean [9]. Citrus black spot infections are more prevalent in the northern provinces of South Africa, where the conditions are hot, with rain in summer, in comparison to the Western Cape province, which has a Mediterranean climate, with cold wet winters and no reported infection [9]. The disease causes cosmetic lesions on the fruit’s rind, rendering the fruit unmarketable. Most citrus fruit varieties are susceptible, to some degree, to infection by the pathogen [10]. Due to CBS, the South African citrus industry risks losing access to the European market. The control of CBS is primarily reliant on the application of fungicidal sprays early in the season, when the fruit is most susceptible to infection [11]. The use of synthetic fungicides has raised concerns due to their toxicity, pollution of the environment, and antimicrobial resistance [12,13]. Therefore, new effective and eco-friendly products are needed for disease management in the citrus industry. The control of phytopathogens has become more challenging, as the pathogens develop resistance as a result of the repeated use of fungicides [12]. Plants produce a diverse range of secondary metabolites in protection of themselves and in communication, while having no specific plant functions or obvious primary purpose [14]. One of the most important classes of plant secondary metabolites is volatile organic compounds (VOCs) [15]. Natural compounds such as secondary metabolites in citrus essential oils and waxes could be viable choices for the development of alternative post-harvest fungicides.

Metabolomics is a valuable tool for significant qualitative and quantitative analyses of complex mixtures with a low molecular weight, primarily from biological samples. It is reliant on gas chromatography coupled to mass spectrometry (GC-MS), high-pressure liquid chromatography coupled to mass spectrometry (HPLC-MS) and nuclear magnetic resonance (NMR) [16]. Metabolomics has established itself as one of the most significant breakthroughs, in pathogens, plants and mammals profiling [17]. Metabolite profiling has been shown in studies to be effective in the separation and identification of targeted and non-targeted plant metabolites, as well as in the classification of numerous plant types and fungi [18].

The metabolomics approach has also been successfully applied in predicting disease tolerance differentiation in citrus varieties, such as the analysis of wild-type orange and the identification of citrus novel natural product enzymes [19,20]. In this study, the analysis of secondary metabolites from the essential oils and cuticular waxes of two citrus cultivars with a varying susceptibility to CBS infection was conducted. A multivariate data analysis was employed to identify essential oil and cuticular wax biomarkers that play a role in CBS susceptibility. The antifungal properties of the identified biomarker compounds against CBS were evaluated in vitro.

## 2. Results and Discussion

### 2.1. Essential Oil Yield at Different Developmental Stages

**Figure 1:**
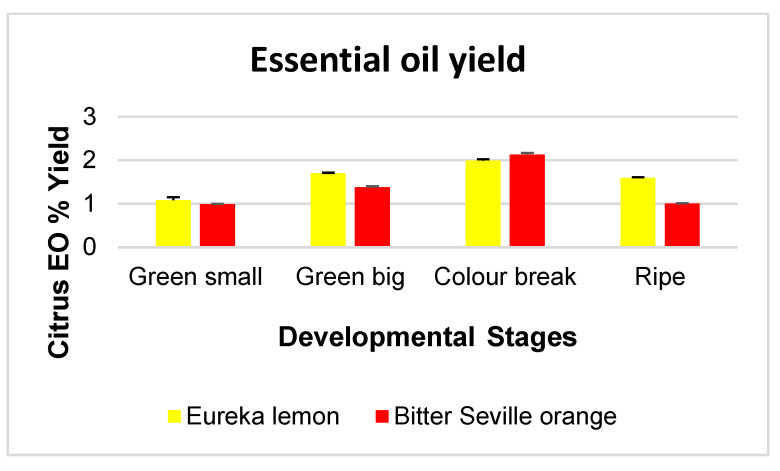
The essential oil yield, for the 2017/2018 season, of Eureka lemon and Bitter Seville orange at various developmental stages.

Four stages of the citrus cultivars were hydro-distilled; green small (1.5 cm), green big (±3 cm), colour break and mature fruits, with yields ranging from 1.08 to 1.99% (*w*/*w*) for Eureka lemon fruit, and 0.99 to 1.38% (*w*/*w*) for Bitter Seville fruit, respectively. The yield of essential oil varied between the cultivars as well as the developmental stage of the fruit, as demonstrated in Figure 1. The essential oil yield of both cultivars increased as the season progressed, reaching the highest levels at the colour break, after which the production of essential oils decreased. Eureka lemons had slightly higher yields of essential oils than Bitter Seville oranges, except at the colour break stage, where the Bitter Seville had somewhat higher levels. The essential oil yield was not repeated for the second season because several studies corresponded with our analysis. The essential oil yield of other citrus cultivars harvested at various developmental stages has been studied, and the same trend has been observed [21]. A study by Esfahani et al., 2017, also indicated variations in essential oil production in *Citrus aurantifolia* as the fruit develops, where the highest essential oil levels were observed in immature fruit (1.54%), with a decrease in semi-mature fruit (0.88%), before increasing again in mature fruit (1.23%) [21]. A study by Wu et al. [22] on *Citrus medica* also indicated an increase in essential oil production as the fruit developed; however, the essential oil yields increased with fruit maturity, and the highest yields were obtained from fully mature fruits.

The decline in yield at maturity can be explained by taking into account the morphological changes of developing fruits associated with biological and structural changes [23]. Genetic factors, plant origin, harvesting and storage practices, developmental stages, techniques of extraction and analysis can all cause variations in essential oil yields [23].

### 2.2. Gas Chromatography–Mass Spectrometry

Chromatographic data revealed that higher levels of metabolites were produced at the early developmental stages, when compared to mature fruit stages, for both essential oil and wax extracts. Young fruits are highly susceptible to *P. citricarpa* infection, primarily taking place within 16 to 28 weeks after flowering [24]. However, disease development accelerates with fruit maturity [25], and therefore higher levels of secondary metabolites may be an indication of natural plant defence mechanisms against possible infection [14,26].

It was clear from the volatile chromatographic data obtained that there are variations in the chemical profile between the different cultivars as well as the developmental stages, Table 1. Bitter Seville, the cultivar with the lowest susceptibility to CBS infection, produced more compounds (16 of the 22 identified compounds) at higher levels at the immature fruit stage than the more susceptible cultivar. Ten of the 23 identified compounds found in Eureka lemons were produced at higher levels at an early developmental stage. This indicates that essential oil composition may play a role in resistance to the pathogen.

**Figure 2:**
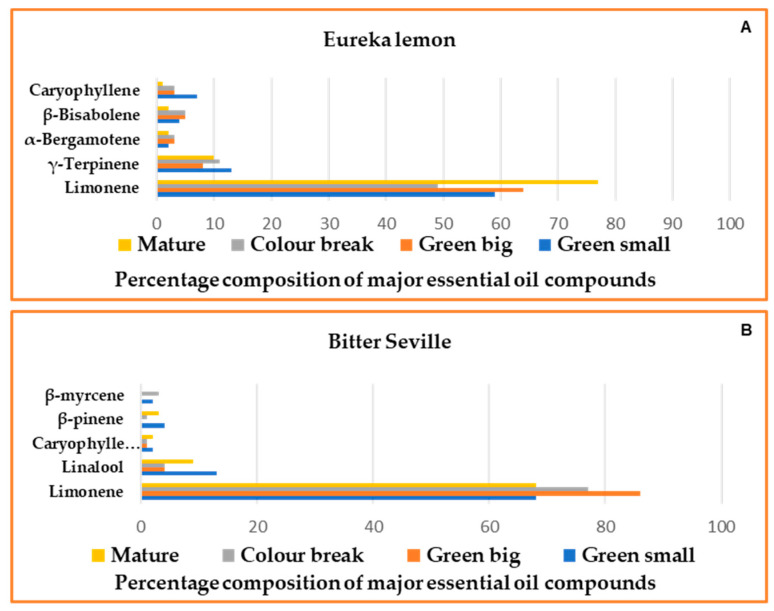
Percentage composition of major essential oil compounds identified in the 2017/2018 season (**A**): Eureka lemons and (**B**): Bitter Seville oranges.

Table 1 lists the percentage levels of all the identified volatile compounds in the two cultivars over the four developmental stages for the 2017/2018 season. The Eureka lemon and Bitter Seville fruit volatiles were primarily composed of monoterpene hydrocarbons, with limonene as the major constituent. The limonene percentage of the total oil ranged from 49 to 77% in Eureka lemons, and 68 to 86% in Bitter Seville, depending on the developmental stage of the fruit. Major secondary metabolites in Eureka lemons included γ-terpinene, caryophyllene, β-bisabolene and α-bergamotene, Figure 2A. Caryophyllene and β-bisabolene were identified in Bitter Seville but at very low levels compared to the levels in Eureka lemons, Table 1. Furthermore, sesquiterpene hydrocarbons were the second most abundant class, with oxygenated monoterpenes being the least abundant. The secondary constituents in Bitter Seville oranges included linalool, caryophyllene, β- pinene and myrcene, Figure 2B. These observations are consistent with those of Vekiari et al. [27], who identified the primary components of citrus essential oils as limonene, β- pinene, myrcene and caryophyllene. Linalool, the biomarker associated with resistance/tolerance (13%), as described in Section 2.3, was detected at much lower levels (2.2%) in the susceptible cultivar. The second highest class observed in Bitter Seville was oxygenated monoterpenes, and the remaining fractions were mainly sesquiterpene hydrocarbons. The second season, 2018/2019, demonstrated the same trend in the volatile compounds produced as observed for the first season.

**Figure 3:**
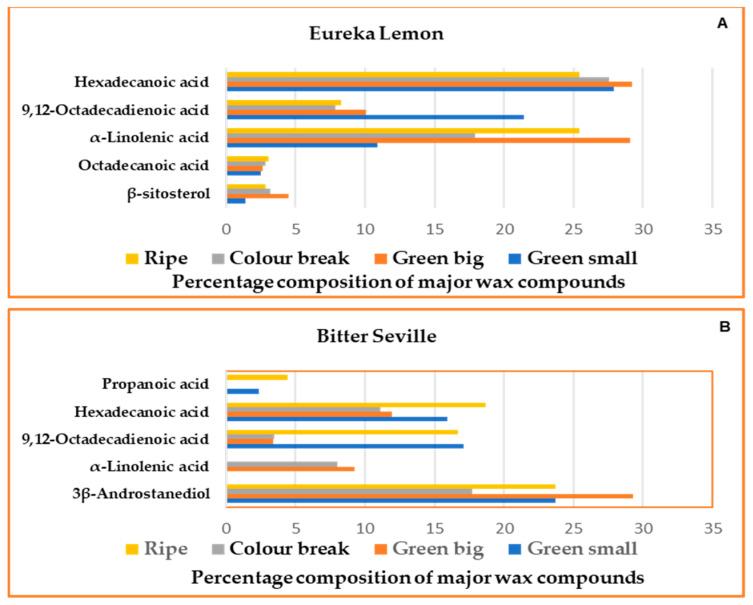
Percentage composition of major wax compounds identified in the 2017/2018 season (**A**): Eureka lemons and (**B**): Bitter Seville oranges.

The wax composition varied between the two cultivars and differed within a cultivar depending on the growth stage, Table 2. Eureka lemon had higher levels of α-linolenic acid and hexadecanoic acid, while Bitter Seville oranges contained a high concentration of androstane-3,17-diol, a compound not found in Eureka lemon. Figure 3 demonstrates the variation between the percentage levels of five compounds detected in the wax extract of both cultivars in the 2017/2018 season. It has been demonstrated that the wax composition varies between plant species and between cultivars within a species [28].

Eureka lemon had high levels of total fatty acids while Bitter Seville oranges contained more aliphatics, alcohols and total wax levels. These observations are in line with those obtained in a study by Wang [29] where aldehydes, alkanes, and fatty acids made up the majority of the surface wax components in mature citrus fruits. The second season 2018/2019 demonstrated the same trend in wax component levels as observed for the first season.

The metabolomic profiles indicated differences in the chemical composition of the volatile and wax extracts of the two cultivars. A metabolomic data analysis would aid in identifying specific metabolites that play a role in the resistance/susceptibility of the cultivars to *P*. *citricarpa.*

### 2.3. Multivariate Data Analysis

#### 2.3.1. Essential Oils Analysis

Principal component analysis (PCA) was used to identify patterns in the chromatography data obtained from the EO and waxes from the two citrus cultivars. All chromatographic data obtained from both seasons and all developmental stages were used to construct the chemometric models. The models were validated using a seven-fold cross-validation analysis, where the Q^2^ value represents the average predicted outcomes across the seven rounds. In addition, sixteen data points from the citrus cultivars were excluded from the model set to use as a prediction set. Pareto scaling was used to construct a model for the citrus varieties using the remaining work set (N = 242). When comparing Eureka lemon (highly susceptible) and Bitter Seville (tolerant), the PCA could not separate the cultivars according to their volatile profiles, indicating a similar chemical composition. A poor PCA model was obtained (R^2^X = 0.439, Q^2^ = 0.212) as a result of the small differentiation between the essential oil components of these cultivars.

**Figure 4:**
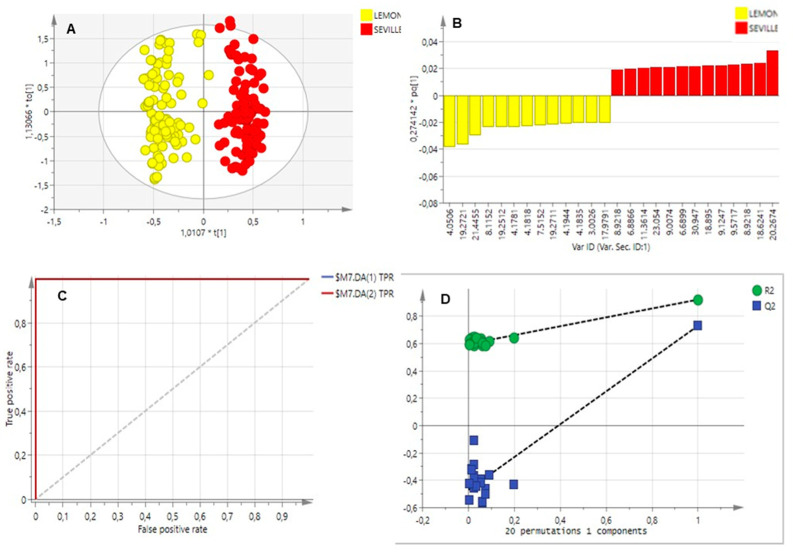
OPLS-DA plots of chromatographic data of volatile metabolite profiling: (**A**): Scores plot demonstrating the separation of the citrus cultivars, Eureka lemon (yellow) and Bitter Seville (red). (**B**): The loadings column plot displays the compounds with the highest contribution to the separation, marked by retention time. The compounds for Bitter Seville can be seen on the right, and Eureka Lemon on the left. (**C**): ROC plot and (**D**): permutation plot.

The chromatographic data of the two cultivars were subsequently grouped into two classes according to susceptibility to the pathogen, to build a supervised OPLS-DA model. An acceptable model was obtained (R^2^Y (cum) = 0.921, indicating high reliability and Q^2^Y = 0.730, indicating high predictability). This model identified compounds that could explain the difference in CBS infection between the two cultivars. The OPLS-DA scores scatter plot is displayed in Figure 4A, and a clear separation of the cultivars can be seen. The orange cultivar, Bitter Seville, clusters to the right of the plot, and the more susceptible cultivar, Eureka lemon, is to the left.

This separation corresponds to the phytochemical difference observed between Eureka lemon and Bitter Seville oranges. The supervised OPLS-DA scores plot separated the cultivars according to chemical compositional and concentration level differences, as shown by the separated clusters in Figure 4A.

The OPLS-DA loadings plot, Figure 4B, identified compounds that contribute towards the separation of the citrus cultivars. This plot visualises the relationship between the metabolites and the model class assignment. This correlation is determined by observing the contribution of the variables on the loadings plot, where variables at the extreme ends of the loadings are highly reliable in discriminating the two groups and used to extract putative biomarkers.

The ROC plot, Figure 4C, and the permutation curve, Figure 4D, validated the model’s predictive capability. The ROC plot displays the true positive classification rate against the false positive classification rate. The area under the curve value, 1.0, indicated a reliable classification of the metabolites associated with resistance or tolerance. In the permutation plot, all Q^2^ values from the data set to the left were lower than the Q^2^ values from the actual data set to the right, and the regression line has a negative *Y*-axis intercept value. The model reliability was confirmed by a CV-ANOVA test where *p* < 0.05; a value of very close to 0 was obtained that it is rounded to 0.

The biomarkers with a possible susceptibility or tolerance to citrus black spot were identified based on retention times and *m/z* values, using the NIST 5 mass spectral library. The loadings plot of susceptibility versus tolerance (Figure 4B) identified linalool oxide (8.921 min), (+)-4-carene (9.572 min), caryophyllene, (18.624 min) and β-cubebene (20.267 min) as possible biomarkers associated with the tolerant Bitter Seville cultivar. The susceptible biomarkers were identified as β-pinene (4.051 min), γ-terpinene (8.115 min), trans-α-bergamotene (19.270 min) and β-bisabolene (21.446 min).

#### 2.3.2. Cuticular Wax Analysis

Principal component analysis (PCA) was also used to determine patterns in the chemistry of very long-chain aliphatic compounds extracted from the two citrus cultivars. The model parameters, an R^2^X of 0.899 and a Q^2^ value of =0.726, indicate a reliable model with good predictive ability.

**Figure 5:**
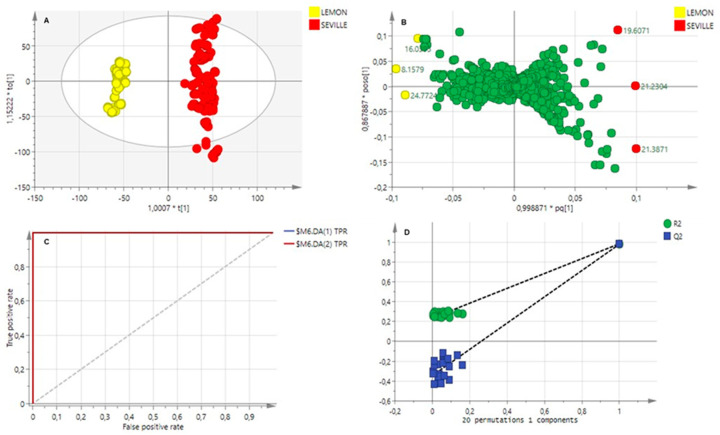
OPLS-DA plots of chromatographic data of wax metabolites. (**A**): Scores plot demonstrating the separation of two citrus cultivars, Eureka lemon marked with yellow (left), and Bitter Seville with red (left). (**B**): Loadings scatter plot with the compounds with the highest contribution to the separation marked with retention time. The separation for Eureka lemon can be seen on the left and Bitter Seville on the right, (**C**): ROC plot and (**D**): permutation plot.

A supervised OPLS-DA model was used to investigate the compounds or classes of compounds that explained the variability observed. Figure 5A depicts the OPLS-DA score plot with the model parameters R^2^Y (cum) = 0.983 and Q^2^Y = 0.978.

In metabolomics studies, OPLS-DA scores plots are frequently used to provide an overview of the data [30,31]. The OPLS-DA scores plot, Figure 5A, clearly indicates the clustering of the two citrus cultivars. The two susceptible citrus cultivars, Eureka lemon, cluster to the left of the plot and the tolerant cultivar, Bitter Seville, to the right. This plot indicates that the two cultivars contained inherent phytochemical compositional differences.

Using the OPLS-DA loadings plot, Figure 5B, the compounds with the highest contribution towards the separation of the citrus cultivars were identified. The loadings plot indicated variables (compounds) that correlated to a high contribution towards the separation of Bitter Seville in red and Eureka lemon in yellow. The biomarkers with possible susceptibility or resistance to citrus black spot were identified based on retention times and *m/z* values, using the NIST mass spectral library.

The loadings plot, Figure 5B, identified octadecanoic acid (19.607 min), androstane-3,17-diol (21.2304 min), and propanoic acid (22.3984 min) as biomarkers associated with resistance. Androstane-3,17-diol was identified as the major compound in Bitter Seville wax extract, and propanoic acid was identified as a minor compound. The susceptible biomarkers were identified as (6.093 min) 2-butenoic acid, (8.1579 min) benzoic acid and (24.772 min) hexadecane.

The same predictive capabilities, ROC and permutation plots, Figure 5C,D, were observed for the volatile data. This indicates a reliable classification of the metabolites associated with resistance or tolerance. The model reliability was confirmed by the CV-ANOVA test, where a value of 0 was obtained (*p* < 0.05).

Androstane-3,17-diol was found at higher levels in mature fruit than in young fruit. Propanoic acid was present at low levels throughout all developmental stages.

### 2.4. In Vitro Investigation of the Biochemical Activity of Secondary Metabolites against the Pathogen

The antifungal activity of the identified biomarker compounds against *P. citricarpa* was evaluated in vitro. According to several studies, radial growth measurement is the preferred method to evaluate the fungicidal qualities of compounds by the indirect measurement of fungal development on solid media [32,33].

Two biomarkers, linalool and caryophyllene, identified from the Bitter Seville volatile extracts were tested for the inhibition of the pathogen. Linalool completely inhibited the pathogen growth at 2000 mg/L. Caryophyllene exhibited little activity against the pathogen, with only a 48.87% inhibition at 2000 mg/L.

In this study, linalool completely inhibited the citrus black spot pathogen at 2000 mg/L. This compound has been previously reported to have some antifungal activities against other citrus pathogens. Linalool demonstrated high antifungal activity against *Citrus acutatum* at doses higher than 100 mg/L [34]. In another study, it was found that in tangerines, limonene and myrcene at a dose of 130 mg/L were not able to inhibit the in vitro germination of conidia of *Alternaria alternata* [35]. However, linalool demonstrated antifungal activity against *A. alternata*, inhibiting more than 97% of the germination of conidia at a similar dose [35]. The current study suggests that the presence of linalool in Bitter Seville orange may be associated with the resistance to infection caused by *P. citricarpa.*

Propanoic acid, androstane-3,17-diol and octadecanoic acid were all identified in the chemometric analysis as wax biomarkers with potential tolerance to citrus black spot. However, the efficacy of androstane-3,17-diol against the pathogen could not be studied due to the unavailability of the standard. The antifungal property of propanoic acid and octadecanoic acid against the pathogen was studied in vitro. Propanoic acid completely inhibited pathogen growth at 750 mg/L, while octadecanoic acid was not effective at controlling the pathogen at all concentrations tested.

Propanoic acid has previously been found to influence membrane permeability and acidify the cytoplasm [36], promoting oxidative stress and apoptosis [37,38,39]. This range of propanoic acid effects on the cell indicates that this compound may target a central regulator of cellular homeostasis or numerous cellular processes at the same time [40]. *Aspergillus nidulans* was used as a model organism to investigate the route of action of this short-chain fatty acid. The main mechanism of action of antifungal fatty acids proposed that fatty acids work by penetrating the lipid bilayers of fungal membranes, impairing the integrity of those membranes and causing an uncontrolled release of intracellular proteins and electrolytes that eventually causes the cytoplasmatic disintegration of fungal cells [41]. According to [42], the antifungal properties of compounds depend significantly on their chemical makeup and the pH of the surrounding environment. The GC-MS fractions of the citrus varieties indicated a variety in length and saturation of fatty acids; therefore, chain length could also have been another factor playing a role in susceptibility to CBS infection. Short-chain length compounds, such as propanoic acid (C3), appear to inhibit the pathogen better than the longer chains such as octadecanoic acid (C18). According to Desbois et al. [43], saturated fatty acids with 10–12 carbon atoms have a higher antimicrobial activity than longer carbon-chain fatty acids [43,44].

## 3. Materials and Methods

### 3.1. Sampling

Fruits of two citrus cultivars, highly susceptible Eureka lemons (*Citrus limon*) and tolerant Bitter Seville (*C. aurantium*), were collected over two seasons, late 2017, 2018 and early 2019, at different developmental stages: small (1.5 cm), big green (±3 cm), colour break and mature fruit. The fruit peels were kept frozen at −18 °C to preserve the metabolites until the time of extraction.

### 3.2. Sample Extraction

#### 3.2.1. Hydro-Distillation of Essential Oils

Citrus peels were washed with water, cut into small pieces, and blended to achieve uniformity using a 450 W Philips blender. The peels (50.0 g) were placed in a 1 L round-bottom flask with 250 mL of water [45] and hydro-distilled at 100 °C for three hours using a Clevenger-type apparatus. Subsequently, 2.00 mL of hexane was used to extract the oils from the distillate and filtered through anhydrous sodium sulphate (Merck, AR grade, Darmstadt, Germany) to remove the water. The samples were kept in amber vials at 4 °C until analysis. Three replicate samples of each of the citrus cultivars at different developmental stages were extracted and analysed.

The percentage yields of Eureka lemon and Bitter Seville orange peels were determined at various developmental stages (green small, green big, colour break and mature), to observe the effect of the developmental stage on the percentage yield using the following equation:

(1) $$\%\;\mathrm{Yield}=\frac{Weight\;of\;oil\;plus\;2.00\;mL\;hexane-Weight\;of\;2.00\;mL\;hexane}{Weight\;of\;fresh\;citrus\;peels}\times$$

#### 3.2.2. Cuticular Wax Extraction

The extraction was performed according to Li et al. [46], with modifications. The peels of the citrus cultivars (Eureka lemon and Bitter Seville) were washed with tap water and air-dried. Seven grams of the chopped citrus fruit rinds was transferred into a glass bottle and 20 mL of chloroform was added, vortexed and then sonicated at 40 °C for 10 min and left to stand for 60 min. Thereafter, the solvent-containing waxes were filtered through 0.5 g of anhydrous sodium sulphate and then dried under nitrogen gas to complete dryness and stored at –20 °C.

### 3.3. Gas Chromatography–Mass Spectrometry Analysis

Prior to GC analysis, all wax extracts were derivatised via reaction with bis-N, N-(trimethylsilyl)-trifluoroacetamide (BSTFA) in pyridine. The method was modified from a derivitisation method described by Kumirska et al., 2013 [47]. The wax extracts were redissolved in 2.0 mL of dichloromethane; subsequently, 10 µL of pyridine was added and incubated at 90 °C for 15 min, followed by the addition of 10 µL of BSTFA containing 1% trimethylchlorosilane (Sigma, Darmstadt, Germany) and incubated at 90 °C for another 15 min. The excess BSTFA was evaporated under nitrogen gas flow, and the samples were dissolved in 2.0 mL dichloromethane for GC-MS analysis.

The chemical composition of the essential oils and major terpenes was determined using an Agilent gas chromatograph (model 7890A, Chemetrix, Johannesburg, South Africa) coupled to a model 5975B mass selective detector and 7693 autosampler. The wax and volatile components were identified by their retention indices, as well as by the NIST version 5 mass spectral library.

The GC-MS parameters for analysing the wax were as follows: A DB-5MS fused silica column (15 m × 0.25 mm id × 0.25 µm), with helium as the carrier gas at a flow rate of 1.0 mL/min. The injection volume was 1.0 µL in splitless mode, with the inlet temperature at 280 °C. The temperature program started at 80 °C, raised at 5 °C/min to 300 °C (held isothermally for 5 min), with a total run time of 49 min.

The parameters for analysing the volatile fractions were as follows: A DB-5MS fused silica column (15 m × 0.25 mm id × 0.25 µm), with helium as the carrier gas at a flow rate of 1.0 mL/min. The injection volume was 1.0 µL in splitless mode, with the inlet temperature at 270 °C. The temperature programme was as follows: An initial temperature of 30 °C for 5 min, raised by 4.5 °C/min to 120 °C (held for 2 min), then raised at 10 °C/min to 275 °C (held for 2 min), then raised at 5 °C/min to 280 °C (held for 1 min), and a total run time of 45.7 min.

The MS parameters for the wax and volatiles were as follows: Acquisition was in scan mode, with a scan range from 55 to 750 amu; electron impact ionisation at 70 eV; ionisation temperature, 230 °C; quadrupole temperature, 150 °C; and the transfer line temperature, 280 °C.

### 3.4. Multivariate Data Analysis

The GC-MS data files were aligned using Marker Lynx^®^ version 4.1 software (Waters Corporation, Milford, MA, USA). The data were pre-processed by spectral alignment, scaling, baseline correction, and noise reduction. The chromatographic data were analysed using SIMCA-P+14.0 software (Metrics, Umeå, Sweden), and Pareto (Par) scaling was applied. Principal component analysis was performed to identify similarities or differences in the chemical composition between the cultivars. OPLS-DA, a supervised method, was used to link the CBS resistance of Seville oranges to chemical constituents [48]. The citrus cultivars were divided into two classes: Class 1 (Eureka Lemon), which is highly susceptible to CBS, and Class 2 (Bitter Seville orange), which is more tolerant to CBS. The OPLS-DA model was validated by obtaining the ROC (receiver operating characteristic) plot, permutation plot and CV-ANOVA (cross-validated analysis of variance). The OPLS-DA S-plots were used to identify putative biomarkers from the two classes of citrus cultivars. The antifungal properties of the identified biomarker compounds were evaluated in vitro.

### 3.5. Antifungal Properties

*Phyllosticta citricarpa* cultures were isolated from CBS-infected fruit, subcultured onto Potato Dextose Agar (PDA) and stored at 4 °C until further use. Carrot agar was prepared according to the method described by Peres et al. [49]. Small plugs (5 mm) of the pathogen were inoculated onto the centre of the Petri plate containing the carrot agar, sealed with parafilm and incubated at room temperature in sunlight for 21 days [35].

The efficacy of the identified essential oil biomarkers (linalool and caryophyllene) and wax biomarkers (propanoic acid, hexadecanoic acid and octadecanoic acid) against the pathogen *P. citricarpa* was determined. Toxic media containing different concentrations (2000 mg/L, 1000 mg/L, 750 mg/L, 500 mg/L and 250 mg/L) of the targeted biomarkers were prepared to identify the minimum inhibitory concentrations. Tween 80 was used as a surfactant (10 μL) to allow the biomarkers to dissolve into the media. Carrot agar containing only Tween was used as a positive control. The radial growth was measured after 21 days, with ten replicate plates per concentration and biomarker used. Radial growth was measured with a KV7150 digital Vernier calliper (Absolute Digimatic-Mitutoyo Corp. Takatsu-ku, Kawasaki, Kanagawa, Japan) for 3 weeks. The antifungal activity of the biomarkers was evaluated as a percentage inhibition of the radial mycelial growth [50].

## 4. Conclusions

This comparative study demonstrated that the essential oil and wax profiles of the two citrus cultivars (Eureka lemon and Bitter Seville) varied depending on the stage of development and amongst cultivars. The chemical profiles of the two cultivars differed, indicating that secondary metabolites play a role in CBS resistance. Bitter Seville had more compounds with higher levels at the green young stage. This is the stage at which infection takes place.

Chemometric models of the chromatographic data of the essential oil and wax extracts were used to identify biomarkers associated with observed resistance in the least susceptible cultivar, Bitter Seville, and the highly susceptible Eureka lemon. Linalool and caryophyllene were identified as biomarkers associated with low susceptibility. The PCA scores plot of the wax chromatographic data indicated separation according to susceptibility to CBS. The OPLS-DA loadings plot identified propanoic acid, androstane-3,17-diol and octadecanoic acid as possible biomarkers of tolerance to citrus black spot. The in vitro efficacy of the identified biomarkers was determined against the pathogen.

The efficacy of the identified compounds against *P. citricarpa* was evaluated in vitro, and linalool completely inhibited pathogen growth at 2000 mg/L; however, at lower levels, complete inhibition was not obtained. Caryophyllene exhibited limited activity (48.87%) against the pathogen at 2000 mg/L. Propanoic acid completely inhibited the pathogen at 750 mg/L. These results indicate that the identified biomarker compounds could protect citrus fruits against *P. citricarpa* infection. The citrus industry should incorporate these compounds in new pest management strategy programs. In the development of new cultivars, attention should be given to the metabolic profiles of the fruit of the new cultivars.

## Figures and Tables

**Table 1:** Chemical components of Eureka lemon and Bitter Seville fruit essential oils obtained at various developmental stages in the 2017/2018 season.

RT	Compound Name	% Relative Content							
		Eureka Lemon				Bitter Seville			
		Green Small	Green Big	Colour Break	Mature	Green Small	Green Big	Colour Break	Mature
2.93	α-pinene	0.80 ± 0.05	0.64 ± 0.08	0.53 ± 0.4	1.3 ± 0.05	1.2 ± 0.7	0.28 ± 0.01	0.67 ± 0.2	0.49 ± 0.1
4.26	β-pinene	0.82 ± 0.3	0.84 ± 0.08	5.2 ± 0.8	2.6 ± 0.7	3.8 ± 2	0.25 ± 0.07	1.1 ± 0.6	3.3 ± 0.4
5.32	β-myrcene	n.d	0.35 ± 0.04	0.29 ± 0.04	0.60 ± 0.1	2.2 ± 1	0.38 ± 0.3	2.69 ± 2	n.d
7.60	Limonene	59 ± 0.6	64 ± 1.2	49 ± 0.7	77 ± 2	68 ± 0.2	86 ± 2	77 ± 2	68 ± 0.01
7.91	Ocimene	n.d	0.030 ± 0.01	0.030 ± 0.01	n.d	3.3 ± 2	n.d	n.d	0.15 ± 2
8.12	γ-Terpinene	13 ± 0.04	7.8 ± 0.3	11 ± 0.3	9.9 ± 2	n.d	n.d	n.d	n.d
8.92	Linalool oxide	n.d	n.d	n.d	n.d	1.9 ± 0.6	0.67 ± 0.4	0.070 ± 2	0.45 ± 2
9.08	cis-Linaloloxide	n.d	n.d	n.d	n.d	0.85 ± 0.2	0.36 ± 0.4	n.d	n.d
9.57	(+)-4-Carene	0.62 ± 1	0.18 ± 0.02	0.48 ± 0.08	0.14 ± 1	n.d	0.090 ± 0.1	0.13 ± 0.05	n.d
10.38	Linalool	4.0 ± 0.03	0.25 ± 0.04	0.20 ± 0.07	0.81 ± 0.01	13 ± 0.2	3.8 ± 0.6	4.4 ± 3	8.5 ± 2
12.64	Terpinen-4-ol	0.68 ± 0.2	1.8 ± 0.1	0.98 ± 0.1	0.38 ± 0.01	0.14 ± 0.2	0.65 ± 0.08	0.47 ± 0.2	0.64 ± 0.02
13.38	α-Terpineol	1.8 ± 0.10	4.4 ± 0.4	1.3 ± 0.1	0.29 ± 0.01	0.25 ± 0.01	0.96 ± 0.5	0.85 ± 0.4	1.1 ± 0.01
14.89	Linalyl acetate	n.d	n.d	n.d	n.d	0.040 ± 0.04	n.d	0.67 ± 0.4	1.0 ± 1
14.65	β-Citral	0.45 ± 0.6	0.010 ± 0.01	0.030 ± 0.01	n.d	n.d	n.d	n.d	n.d
15.70	α-Citral	0.25 ± 0.1	0.030 ± 0.01	0.010 ± 0.05	n.d	n.d	n.d	n.d	n.d
15.98	cis Farnesol	0.090 ± 0.1	0.090 ± 0.8	0.090 ± 0.8	0.040 ± 0.01	n.d	n.d	n.d	n.d
16.01	Camphene	n.d	n.d	n.d	n.d	0.020 ± 0.03	n.d	n.d	n.d
16.29	γ-Elemene	0.54 ± 0.04	0.16 ± 0.05	0.59 ± 0.2	0.29 ± 0.2	0.14 ± 0.06	n.d	0.12 ± 0.01	n.d
16.50	δ-Elemene	n.d	n.d	n.d	n.d	n.d	0.11 ± 0.05	0.44 ± 0.02	0.49 ± 0.4
18.00	β-Elemene	n.d	n.d	n.d	n.d	0.14 ± 0.06	n.d	0.28 ± 0.2	0.30 ± 0.8
18.03	Farnesol acetate	0.13 ± 0.06	0.89 ± 0.1	0.27 ± 0.1	0.090 ± 0.02	n.d	n.d	n.d	n.d
18.62	Caryophyllene	6.9 ± 0.04	2.8 ± 0.1	2.6 ± 0.4	1.1 ± 0.04	1.9 ± 0.7	0.35 ± 0.04	0.93 ± 0.06	1.5 ± 0.60
18.83	Germacrene D	n.d	n.d	n.d	n.d	1.6 ± 1	n.d	0.82 ± 0.5	n.d
19.27	α-Bergamotene	2.4 ± 1	2.7 ± 0.2	2.9 ± 0.3	1.5 ± 0.1	n.d	n.d	n.d	n.d
19.42	α-Caryophyllene	0.67 ± 0.03	0.35 ± 0.05	0.29 ± 0.07	0.16 ± 0.06	0.34 ± 0.2	0.080 ± 0.01	n.d	0.020 ± 0.5
19.82	β-Santalene	0.14 ± 0.08	0.14 ± 0.01	0.14 ± 0.03	0.070 ± 0.01	n.d	n.d	n.d	n.d
20.12	β-Farnesene	0.17 ± 0.01	0.22 ± 0.08	0.18 ± 0.7	0.060 ± 0.2	n.d	n.d	n.d	n.d
20.27	β-Cubebene	n.d	n.d	n.d	n.d	0.61 ± 0.07	0.15 ± 0.01	n.d.	0.88 ± 0.1
20.60	γ-Muurolene	0.02 ± 0.05	0.080 ± 0.66	0.060 ± 0.01	n.d	0.23 ± 0.1	n.d	0.12 ± 0.6	0.22 ± 0.1
21.45	β-Bisabolene	4.0 ± 0.20	4.9 ± 1.13	4.9 ± 0.3	2.3 ± 0.01	0.11 ± 0.2	n.d	0.18 ± 0.08	0.070 ± 0.2
21.48	α-Cadinene	n.d	n.d	n.d	n.d	0.030 ± 0.01	0.070 ± 0.04	n.d	0.21 ± 0.04
21.77	β-Sesquiphellandrene	n.d	n.d	n.d	n.d	0c.10 ± 0.04	n.d	0.11 ± 0.2	0.09 ± 0.3
22.85	Caryophyllene oxide	0.060 ± 0.01	0.16 ± 0.06	0.080 ± 0.01	n.d	n.d	n.d	n.d	n.d
22.97	Spathulenol	0.060 ± 0.01	0.29 ± 0.05	0.25 ± 0.09	0.070 ± 0.06	n.d	n.d	n.d	n.d
24.77	Azulene	n.d	n.d	n.d	n.d	n.d	n.d	n.d	n.d
25.84	cis-α-Bisabolene	0.21 ± 0.08	0.15 ± 0.61	0.28±0.08	0.060 ± 0.02	n.d	n.d	n.d	n.d

Data presented as mean SD (N = 3), n.d = no detection of chemical compounds, RT is Retention Time.

**Table 2:** Chemical constituents of Eureka lemon and Bitter Seville fruit wax obtained at various developmental stages in the 2017/2018 season.

RT	Chemical Formula	Compound Name	%Relative Area							
			Eureka Lemon				Bitter Seville			
			Green Young	Green Big	Colour Break	Ripe	Green Young	Green Big	Colour Break	Ripe
3.57	C_10_H_18_O	Geraniol	0.72 ± 0.2	n.d	1.4 ± 1	n.d	n.d	n.d	n.d	n.d
5.04	C_10_H_16_O_2_	2-Butenoic acid, 3-hexenyl ester	2.3 ± 2	n.d	n.d	n.d	n.d	n.d	0.050 ± 0.3	0.050 ± 0.1
10.84	C_7_H_6_CO_2_	Benzoic acid	n.d	1.5 ± 1	n.d	3.1 ± 1	n.d	n.d	0.77 ± 1	0.65 ± 0.8
12.45	C_14_H_28_O_2_	Tetradecanoic acid	0.23 ± 0.3	0.25 ± 0.2	0.82 ± 1	0.25 ± 0.9	0.17 ± 0.1	0.22 ± 0.2	0.070 ± 0.9	0.24 ± 0.2
12.97	C_8_H_8_O_2_	Benzeneacetic acid	n.d	1.1 ± 0.6	n.d	1.5 ± 0.5	n.d	n.d	n.d	n.d
13.26	C_20_H_42_	Eicosane	n.d	n.d	0.31 ± 0.3	n.d	n.d	n.d	n.d	n.d
14.22	C_15_H_30_O_2_	n-Pentadecanoic acid	1.2 ± 0.06	1.9 ± 1	2.2 ± 0.2	n.d	n.d	n.d	n.d	n.d
15.96	C_16_H_30_O_2_	Hexadecanoic acid	28 ± 0.02	29 ± 1	29 ± 1	25 ± 2	16 ± 0.2	12 ± 0.3	11 ± 1	19 ± 1
16.82	C_9_H_8_O_2_	Cinnamic acid	0.60 ± 0.2	2.5 ± 0.7	n.d	4.1 ± 0.8	n.d	n.d	8.6 ± 1	5.0 ± 2
17.60	C_17_H_34_O_2_	Heptadecanoic acid	n.d	1.2 ± 1	n.d	1.4 ± 1	0.36 ± 0.3	0.31 ± 0.1	n.d	1.3 ± 0.3
17.61	C_10_H_20_O_2_	Decanoic acid	0.53 ± 0.5	n.d	n.d	n.d	n.d	0.61 ± 0.2	n.d	n.d
18.59	C_18_H_32_O_2_	9,12-Octadecadienoic acid (Z,Z)	21 ± 2	10 ± 0.2	7.9 ± 0.8	8.3 ± 1	17 ± 1	3.4 ± 0.9	3.4 ± 2	17 ± 1
18.77	C_18_H_30_O_2_	α-Linolenic acid	12 ± 1	29 ± 0.9	18 ± 0.7	25 ± 1	n.d	9.3 ± 1	8.0 ± 2	n.d
19.26	C_18_H_36_O_2_	Octadecanoic acid	2.5 ± 1	2.6 ± 2	2.8 ± 1	3.1 ± 1	1.7 ± 1	0.34 ± 1	1.4 ± 0.9	2.3 ± 0.8
19.98	C_3_H_4_O_2_	Propanoic acid	n.d	0.18 ± 0.9	1.1 ± 0.9	n.d	2.3 ± 1	n.d	n.d	4.5 ± 1
20.06	C_17_H_36_	Heptadecane	n.d	n.d	0.44 ± 0.7	0.48 ± 0.8	0.36 ± 0.3	0.31 ± 0.1	n.d	1.3 ± 0.3
21.33	C_19_H_32_O_2_	Androstane-3, 17 diol	n.d	n.d	n.d	n.d	25 ± 1	29 ± 1	18 ± 1	24 ± 0.9
21.61	C_24_H_50_	Tetracosane	n.d	n.d	3.1 ± 0.1	n.d	n.d	n.d	n.d	n.d
23.17	C_16_H_34_	Hexadecane	n.d	0.18 ± 1	n.d	n.d	n.d	n.d	n.d	n.d
28.70	C_29_H_60_	Nonacosane	n.d	0.43 ± 0.6	n.d	n.d	n.d	n.d	n.d	n.d
29.94	C_28_H_58_	Octacosane	n.d	n.d	0.89 ± 0.7	0.31 ± 0.9	n.d	0.18 ± 1	0.15 ± 0.8	n.d
31.15	C_18_H_36_	Octadecane	0.20 ± 1	n.d	n.d	0.57 ± 0.8	n.d	n.d	0.070 ± 0.18	0.080 ± 0.3
32.03	C_28_H_48_O	Campesterol	0.25 ± 0.9	1.0 ± 26	0.80 ± 0.1	0.68 ± 0.2	0.16 ± 0.3	0.21 ± 0.3	0.25 ± 0.4	0.14 ± 0.2
32.37	C_29_H_48_O	Stigmasterol	0.89 ± 0.9	1.7 ± 0.3	6.7 ± 0.4	0.85 ± 0.4	0.47 ± 0.3	0.51 ± 0.8	0.41 ± 0.6	0.32 ± 0.9
33.06	C_29_H_50_O	β-Sitosterol	1.4 ± 1	4.5 ± 0.8	3.2 ± 1	2.9 ± 1	0.97 ± 0.3	1.5 ± 0.3	1.1 ± 0.2	0.76 ± 0.4

Data presented as mean SD (N = 3), n.d = no detection of chemical compound, RT is Retention Time.

## Data Availability

The original contributions presented in the study are included in the article, further inquiries can be directed to the corresponding author.
